# Social epidemiology of early adolescent alcohol expectancies

**DOI:** 10.1186/s12889-023-17434-5

**Published:** 2023-12-13

**Authors:** Jason M. Nagata, Gabriel Zamora, Natalia Smith, Omar M. Sajjad, Joan Shim, Kyle T. Ganson, Alexander Testa, Dylan B. Jackson

**Affiliations:** 1 Department of Pediatrics, University of California, San Francisco, 550 16th Street, 4th Floor, Box 0530, San Francisco, CA 94158 USA; 2 Geisel School of Medicine, Dartmouth College, 1 Rope Ferry Rd, Hanover, NH 03755 USA; 3 Factor-Inwentash Faculty of Social Work, University of Toronto, 246 Bloor St W, Toronto, ON M5S 1V4 Canada; 4 Department of Management, Policy and Community Health, University of Texas Health Science Center at Houston, 7000 Fannin St, Houston, TX 77030 USA; 5 Department of Population, Family, and Reproductive Health, Johns Hopkins Bloomberg School of Public Health, Johns Hopkins University, 615 N Wolfe St, Baltimore, MD 21205 USA

**Keywords:** Alcohol, Adolescent, Alcohol expectancies, Substance use, Alcohol sipping

## Abstract

**Purpose:**

To determine the sociodemographic correlates of alcohol expectancies (i.e., beliefs regarding positive or negative effects of alcohol) in a national (U.S.) cohort of early adolescents 10–14 years old. A second aim was to determine associations between alcohol sipping and alcohol expectancies.

**Methods:**

We analyzed cross-sectional data from the Adolescent Brain Cognitive Development (ABCD) Study (N = 11,868; Year 2). Linear regression analyses were conducted to estimate associations between sociodemographic factors (sex, race/ethnicity, sexual orientation, household income, parental education, parent marital status, religiosity) and positive (e.g., stress reduction) and negative (e.g., loss of motor coordination) alcohol expectancies. Additional linear regression analyses determined associations between alcohol sipping and alcohol expectancies, adjusting for sociodemographic factors.

**Results:**

Overall, 48.8% of the participants were female and 47.6% racial/ethnic minorities, with a mean age of 12.02 (SD 0.67) years. Older age among the early adolescent sample, male sex, and sexual minority identification were associated with more positive and negative alcohol expectancies. Black and Latino/Hispanic adolescents reported less positive and negative alcohol expectancies compared to White non-Latino/Hispanic adolescents. Having parents with a college education or greater and a household income of $200,000 and greater were associated with higher positive and negative alcohol expectancies. Alcohol sipping was associated with higher positive alcohol expectancies.

**Conclusions:**

Older age, White non-Latino/Hispanic race, male sex, sexual minority status, higher parental education, and higher household income were associated with higher positive and negative alcohol expectancies. Future research should examine the mechanisms linking these specific sociodemographic factors to alcohol expectancies to inform future prevention and intervention efforts.

**Supplementary Information:**

The online version contains supplementary material available at 10.1186/s12889-023-17434-5.

## Background

Previous literature has defined alcohol expectancies as beliefs regarding the positive or negative effects of alcohol [1]. Expectancy theory centers around these beliefs that drinking alcohol can have positive (e.g., stress relief) and negative (e.g., loss of motor coordination) effects [2]. Positive alcohol expectancies in adolescents have consistently been found to be predictive of future alcohol use [3, 4]. Negative alcohol expectancies were previously associated with less drinking [5]; however, more recent studies have shown negative alcohol expectancies to be associated with problematic drinking [3, 6]. Individuals with negative alcohol expectancies may influence their behavior in a manner that self-fulfills the negative effects they expect. The psychological concept of self-fulfilling prophecy was demonstrated in previous studies showing college students with more positive expectancies were more likely to report experiencing those same consequences as a result of their alcohol use that day [7]. This held true for students with more negative expectancies, such as becoming aggressive or embarrassing themselves [7]. Adolescent alcohol use has been associated with altered attention and memory, increased anxiety, and risk-taking behaviors [8]. Risky health behaviors like alcohol use are often initiated during adolescence [9]. Understanding the early correlates of alcohol expectancies in early adolescence is valuable given the role expectancies play in the development of later problematic drinking behaviors. Identifying sociodemographic factors such as sex, race/ethnicity, household income, parent education, and parent marital status will allow for the development of preventive interventions and public health programs.

There is currently limited literature examining the sociodemographic factors associated with alcohol expectancies in adolescence. Previous literature often focused on one sociodemographic factor and its associations with alcohol expectancies but did not focus on early adolescents. For example, previous research has found that men have greater positive and negative alcohol expectancies than women [10, 11]. Race and ethnicity and their associations with alcohol expectancies have been analyzed in 12–18-year-old participants. The studies found that while African-American youth initially had higher positive alcohol expectancies relative to White youth, more positive alcohol expectancies were only predictive of alcohol use in White youth [12, 13]. However, another study in the United Kingdom found that White British children had higher positive alcohol expectancies than Black and Asian British children [14]. Several studies have found that older age is associated with higher positive expectancies [14–16]. The current literature has also established positive associations between socioeconomic status and adolescent alcohol use [17]. As with socioeconomic status, higher parental education has been associated with more positive expectancies [14]. Other parental factors such as parental divorce have been associated with increased risk for adolescent drinking onset [18]. Religiosity has been associated with higher negative expectancies [19]. While previous literature has identified alcohol expectancies as a predictive risk factor for alcohol abuse in adult sexual minorities, there is currently scant literature analyzing the associations between sexual orientation and alcohol expectancies in early adolescents [20]. However, previous studies have found that sexual minority adolescents engage in alcohol use earlier and with steeper drinking trajectories into young adulthood relative to heterosexual youth [21].

One analysis of the Adolescent Brain Cognitive Development Study found that older age (within the 10-14-year-old age range) was associated with greater positive and negative alcohol expectancies in linear mixed-effects models [22]. Compared to non-Hispanic Black or Hispanic ethnicity, non-Hispanic white ethnicity was associated with higher positive and negative alcohol expectancies [22]. Male sex was associated with higher negative alcohol expectancies [22]. However, the study did not examine household income, parent education, parent marital status, religiosity, or sexual orientation associations with alcohol expectancies. Furthermore, the study speculated that previous alcohol experience (e.g., alcohol sipping) may impact alcohol expectancies, which was identified as an area of future research [22].

The breadth of literature has been useful in identifying possible sociodemographic factors for exploration in their associations with adolescent alcohol expectancies. However, the current literature has not targeted early adolescents nor focused on a large array of sociodemographic factors. The current study aims to bridge this gap in the literature by first examining the relationship between several sociodemographic characteristics and their associations with alcohol expectancies in a demographically diverse national cohort of early adolescents. Male sex, White non-Latino/Hispanic race/ethnicity, sexual minority status, higher parent education, and non-religiosity were hypothesized to be associated with greater positive and negative alcohol expectancies. Secondly, the study aimed to determine the relationship between alcohol sipping and alcohol expectancies. Alcohol sipping was hypothesized to be associated with greater alcohol expectancies.

## Methods

Cross-sectional data from 2018 to 2020 (Year 2, 4.0 data release) of the Adolescent Brain Cognitive Development (ABCD) Study were analyzed. The ABCD Study® is the largest longitudinal study of health and brain development in American youth. At baseline (2016–2018), the study began following a cohort of 11,878 9-10-year-olds from 21 racially/ethnically diverse sites within the U.S. (Year 0). Further details about the ABCD Study recruitment, participants, protocol, and measures have been previously described [23]. Data analyzed in this study were collected by adolescents’ and parents’ reports at Year 2 (mean adolescent age 12.02 [SD 0.67] years, range 10–14 years) of the ABCD assessment. There was missing data for sociodemographic factors and alcohol expectancies among 2,863 participants. Comparisons of those missing versus not missing data are shown in Table S1; adolescents without missing data were more likely to be White non-Latino/Hispanic, have a household income of $75,000 and greater, have parents with higher education, and have parents who were married/partnered. The University of California, San Diego (UCSD) provided centralized institutional review board (IRB) approval while study sites obtained approval from their local IRBs. Caregivers provided written informed consent, and each adolescent provided written assent.

## Measures

### Dependent variables: alcohol expectancies

Alcohol expectancies data were collected by adolescents’ self-reported agreement/disagreement with statements about the effects of alcohol on a 5-point Likert scale ranging from 1 to 5, through the Alcohol Expectancy Questionnaire- Adolescent, Brief (AEQ-AB) [24]. The AEQ-AB questionnaire is a 7-item measure comprised of 2 component mean scores: positive alcohol expectancies (4 items) and negative alcohol expectancies (3 items). Having positive alcohol expectancies often includes beliefs that alcohol increases sociability, reduces tension, and increases sexual enjoyment. One could also have negative alcohol expectancies, or a belief that alcohol causes cognitive impairment, risky behavior, and aggression. Both positive and negative alcohol expectancies are associated with alcohol use [25]. Examples include, “Alcohol can help how well a person gets along with others (makes people want to have fun together)” (positive) and “Alcohol can hurt how well a person gets along with others (makes people mean to others)” (negative). See Table S2 for a full list of the questions and responses. Average scores on a particular component (positive alcohol expectancies; negative alcohol expectancies) were calculated as suggested in the literature and analyzed based on principal components (positive and negative alcohol expectancies) [24]. Cronbach alphas were 0.69 for positive alcohol expectancies and 0.69 for negative alcohol expectancies in the current analysis. The correlation between the two subscales was − 0.01.

### Independent variables: sociodemographic characteristics

One parent/guardian per adolescent (85.3% mothers, 10.0% fathers, 2.3% adoptive parent, 1.0% custodial parent, 1.4% other) completed a demographic survey about the adolescent. Sex, race/ethnicity, and child religiosity were assessed at the baseline parent survey. Household income, highest parent education, and parent marital status were assessed at the Year 2 parent survey. More detailed descriptions of the questions, response options, and coding are shown in Table S3.

#### Sex

Parents reported the sex assigned at birth of the child (female or male).

#### Race/ethnicity

A single race/ethnicity variable was constructed based on parent report at baseline, following guidance from the National Longitudinal Study of Adolescent to Adult Health: White non-Latino/Hispanic, Latino/Hispanic, Black non-Latino/Hispanic, Asian non-Latino/Hispanic, Native American non-Latino/Hispanic, and other non-Latino/Hispanic [26].

#### Household income

Income at Year 2 was categorized into six categories based on parent report: less than $25,000, $25,000 through $49,999, $50,000 through $74,999, $75,000 through $99,999, $100,000 through $199,999, or $200,000 and greater.

#### Highest parent education

Parents reported in Year 2 the highest level of education of the parent or partner (highest of the two). Response categories were combined into high school or less and college education or more.

#### Parent marital status

Parents were asked in Year 2 their marital status. Married and living with a partner were combined. Widowed, divorced, separated, and never married were categorized as not married/not living with a partner.

#### Sexual orientation

Participants were asked in Year 2, “Are you gay or bisexual?” Response options included: yes, maybe, no, and don’t understand the question.

#### Child religiosity

Parents were asked at baseline, “What is the child’s religious preference?” Responses were combined into religious (Mainline Protestant, Evangelical Protestant, Historically Black Church, Roman Catholic, Jewish, Mormon, Jehovah’s Witness, Muslim, Buddhist, Hindu, Orthodox Christian, Unitarian, Other Christian) and not religious (Atheist, Agnostic, Something else, Nothing in Particular).

#### Alcohol sipping

Adolescents in the ABCD study completed an adapted version of the Timeline Follow-Back (TLFB) instrument, which is a retrospective, calendar-based self-report instrument for measuring the use of alcohol and other addictive substances [27, 28]. This instrument exhibits high test-retest reliability as well as discriminant and convergent validity with other measures [29]. The instrument has been validated in adolescents [30, 31] and prior studies have demonstrated the reliability and validity of self-reported data on alcohol use in children [32, 33]. The current analysis used the question of whether children had tried a sip of alcohol such as beer, wine, or liquor (rum, vodka, gin, whiskey) at Year 2.

### Statistical analyses

Statistical analyses were performed using Stata 18 (StataCorp) and R Studio. For the first aim, multiple linear regression analyses were conducted to estimate associations between sociodemographic factors (age, sex, race/ethnicity, sexual orientation, household income, parental education, parent marital status, religiosity) and alcohol expectancies (separate regressions for positive and negative expectancies). For the second aim, multiple linear regression analyses were conducted to estimate associations between alcohol sipping and alcohol expectancies (separate regressions for positive and negative expectancies), adjusting for age, sex, race/ethnicity, sexual orientation, household income, parental education, parent marital status, and religiosity. Linear regression coefficients were standardized to β coefficients. Propensity weights were applied to match key sociodemographic variables in the ABCD Study to the American Community Survey from the U.S. [34]. The two-sided alpha was set at 0.05. To address missing data, we applied multiple imputation with chained equations using the *mice* package in R, with 10 imputed datasets formed with a final sample size of 11,868 [35].

In supplemental analyses, the prior linear regression analyses estimating the associations between sociodemographic factors and alcohol expectancies were stratified by never sipped alcohol versus sipped alcohol. Effect modification of sociodemographic factors by alcohol sipping on alcohol expectancies was tested.

## Results

Table 1 describes sociodemographic characteristics among the adolescents of the ABCD Study who met the inclusion criteria (N = 11,868). 48.8% of the participants were female and 47.6% were racial/ethnic minorities. Mean positive and negative alcohol expectancies were 1.97 (SD = 0.79) and 4.11 (SD = 0.93), respectively.

**Table 1:** Sociodemographic and alcohol expectancies characteristics of Adolescent Brain Cognitive Development (ABCD) Study participants (N = 11,868)

Sociodemographic characteristics	Mean (SD)
Age (years)	12.02 (0.67)
Sex (%)	
Female	48.8%
Male	51.2%
Race/ethnicity (%)	
White non-Latino/Hispanic	52.4%
Latino / Hispanic	20.1%
Black non-Latino/Hispanic	17.3%
Asian non-Latino/Hispanic	5.5%
Native American non-Latino/Hispanic	3.2%
Other non-Latino/Hispanic	1.5%
Sexual Orientation Survey (%)	
No	87.4%
Yes	4.4%
Maybe	3.8%
Don’t understand the question	3.2%
Decline to answer	1.3%
Household income (%)	
Less than $75,000	47.6%
$75,000 and greater	52.4%
Parents’ highest education (%)	
High school education or less	16.2%
College education or more	83.8%
Parents’ marital status (%)	
Married/partnered	69.8%
Not married/unpartnered/single	30.2%
Religiosity (%)	
Religious	74.6%
Not religious	25.4%
Alcohol Expectancies	
Positive	1.97 (0.79)
Negative	4.11 (0.93)
A sip of alcohol such as beer, wine, or liquor (rum, vodka, gin, whiskey)	
No	90.2%
Yes	9.8%

ABCD propensity weights were applied based on the American Community Survey from the US Census. SD = standard deviation

Table 2 shows multiple linear regression models examining sociodemographic correlations with positive and negative alcohol expectancies in the total sample. Each additional year of life among the early adolescent sample was associated with more positive (β = 0.12 [95% 0.10, 0.14]) and negative (β = 0.03 [95% 0.01, 0.05]) alcohol expectancies. Black non-Latino/Hispanic adolescents reported less positive (β = -0.12 [95% -0.19, -0.05]) and negative (β = -0.10 [95% -0.16, -0.04]) alcohol expectancies relative to White non-Latino/Hispanic adolescents. Similarly, Latino/Hispanic adolescents showed less positive (β = -0.10 [95% -0.16, -0.03]) and negative (β = -0.09 [95% -0.16, -0.02]) alcohol expectancies compared to White non-Latino/Hispanic adolescents. Sexual minority adolescents reported higher positive (β = 0.31 [95% 0.21, 0.41]) and negative (β = 0.14 [95% 0.04, 0.24]) alcohol expectancies compared to heterosexual peers. Adolescents who said “maybe” to the sexual minority questions reported higher positive (β = 0.27 [95% 0.17, 0.37]) alcohol expectancies compared to heterosexual peers. In general, a household income of $200,000 and greater was associated with higher positive and negative alcohol expectancies compared to lower income groups. Having parents with a high school education or less was associated with less positive (β = -0.12 [95% -0.18, -0.06]) and negative (β = -0.21 [95% -0.28, -0.15]) alcohol expectancies compared to having parents with a college education or greater. Reporting no religiosity was associated with higher positive (β = 0.14 [95% 0.09, 0.18]) but not negative alcohol expectancies compared to reporting religiosity.

**Table 2:** Sociodemographic associations with positive and negative alcohol expectancies in the Adolescent Brain Cognitive Development (ABCD) Study (N = 11,868)

	Positive Alcohol Expectancies, Adjusted		Negative Alcohol Expectancies, Adjusted	
Sociodemographic characteristics	β (95% CI)	*p*	β (95% CI)	*p*
Age (years)	**0.12 (0.10, 0.14)**	**< 0.001**	**0.03 (0.01, 0.05)**	**0.003**
Sex				
Female	reference		reference	
Male	**0.04 (0.00, 0.09)**	**0.043**	**0.07 (0.03, 0.11)**	**0.002**
Race/ethnicity				
White non-Latino/Hispanic	reference		reference	
Latino / Hispanic	**-0.10 (-0.16, -0.03)**	**0.003**	**-0.09 (-0.16, -0.02)**	**0.012**
Black non-Latino/Hispanic	**-0.12 (-0.19, -0.05)**	**< 0.001**	**-0.10 (-0.16, -0.04)**	**0.002**
Asian non-Latino/Hispanic	-0.07 (-0.18, 0.03)	0.142	-0.01 (-0.12, 0.09)	0.803
Native American non-Latino/Hispanic	-0.10 (-0.21, 0.02)	0.117	-0.10 (-0.21, 0.02)	0.090
Other non-Latino/Hispanic	-0.10 (-0.26, 0.06)	0.235	-0.10 (-0.27, 0.07)	0.253
Sexual minority status				
No	reference		reference	
Yes	**0.31 (0.21, 0.41)**	**< 0.001**	**0.14 (0.04, 0.24)**	**0.006**
Maybe	**0.27 (0.17, 0.37)**	**< 0.001**	0.09 (-0.02, 0.19)	0.113
Don’t understand the question	-0.07 (-0.18, 0.04)	0.219	-0.06 (-0.18, 0.06)	0.312
Decline to answer	0.12 (-0.06, 0.29)	0.193	0.10 (-0.07, 0.28)	0.246
Household income				
$200,000 and greater	reference		reference	
$100,000 to $199,999	**-0.12 (-0.20, -0.03)**	**0.005**	0.00 (-0.09, 0.08)	0.948
$75,000 to $99,999	**-0.10 (-0.19, -0.01)**	**0.024**	-0.04 (-0.13, 0.05)	0.335
$50,000 to $74,999	**-0.14 (-0.22, -0.05)**	**0.003**	**-0.09 (-0.18, 0.00)**	**0.048**
$25,000 to $49,999	**-0.13 (-0.22, -0.04)**	**0.005**	-0.05 (-0.15, 0.04)	0.266
$24,999 or less	**-0.18 (-0.28, -0.07)**	**< 0.001**	**-0.19 (-0.29, -0.09)**	**< 0.001**
Parents’ highest education				
College education or more	reference		reference	
High school education or less	**-0.12 (-0.18, -0.06)**	**< 0.001**	**-0.21 (-0.28, -0.15)**	**< 0.001**
Parents’ marital status				
Married/partnered	reference		reference	
Not married/unpartnered/single	**0.05 (0.00, 0.10)**	**0.042**	0.02 (-0.03, 0.07)	0.495
Child religiosity				
Religious	reference		reference	
Not religious	**0.14 (0.09, 0.18)**	**< 0.001**	0.01 (-0.04, 0.05)	0.772

Bold indicates *p* < 0.05. ABCD propensity weights were applied based on the American Community Survey from the US Census. All models include age, sex, race/ethnicity, sexual orientation, household income, parent education, parent marital status, child religiosity, and study site.

Sipping alcohol was associated with positive (β = 0.35 [95% CI 0.28, 0.42]) but not negative alcohol expectancies, in linear regression models adjusting for sociodemographic variables (Table S4).

Supplemental analyses show multiple linear regression models examining sociodemographic correlations with positive (Table S5) and negative (Table S6) alcohol expectancies, stratified by never sipped alcohol compared to sipped alcohol. In general, most of the sociodemographic associations held within the subsample who had never sipped alcohol. Overall, there were no significant interactions with alcohol sipping and sociodemographic factors for the association with positive alcohol expectancies (Table S7).

## Discussion

The current study contributes new insights and extends previous literature by identifying several sociodemographic factors associated with positive and negative alcohol expectancies in a diverse nationwide sample of early adolescents ages 10–14 years old. Specifically, this study found older age, male sex, White non-Latino/Hispanic race, sexual minority status, higher household income, and higher parent education to be associated with higher positive and negative alcohol expectancies among early adolescents. It is notable that most of the sociodemographic associations with positive and negative alcohol expectancies held within the subsample who had never sipped alcohol. In addition, previous alcohol sipping is associated with higher positive alcohol expectancies. These results are consistent with previous literature [13, 15, 19, 20]; however, the associations between sexual minority status and positive and negative alcohol expectancies as well as lack of religiosity with positive alcohol expectancies are novel in their applicability to early adolescence. Stressors due to sexual minority status have previously been predictive of greater alcohol use [36]. These same stressors related to sexual minority status may also explain the associations with positive and negative alcohol expectancies in this group [36]. Past studies have posited that individuals dealing with minority stressors may use alcohol as a coping mechanism to deal with the discrimination they experience [37]. Thus, adolescents facing minority stressors may develop expectancies that drinking can help them temporarily alleviate stress or escape from their daily struggles. Previous literature has demonstrated associations between sexual identity uncertainty and a higher risk for alcohol use disorder [38]. As for religiosity, church attendance has been shown to serve as a positive influence that reduces the odds of youth’s alcohol use [39]. Lack of traditions around the morality of alcohol without religious affiliation may contribute to the higher positive alcohol expectancies seen in this group. Compared to Latino/Hispanic and Black adolescents, White non-Latino/Hispanic adolescents had higher positive and negative alcohol expectancies. This is consistent with previous literature that found White children to have higher positive alcohol expectancies [12, 13], but the current study also demonstrates White adolescents to have higher negative alcohol expectancies relative to other racial groups.

Regarding parental factors, having parents with a high school education or less was associated with less positive and negative alcohol expectancies. This is again consistent with previous literature as it pertains to positive expectancies [14]. but is novel in terms of its association with lower negative expectancies. Past literature determined that children with more educated parents had higher internalizing problems such as feeling sad or worried, which in turn, predicted higher negative alcohol expectancies [14]. While perceivably counterintuitive that significant associations were found in the same direction for positive and negative expectancies, this finding highlights the complexity of the processes that influence adolescent perceptions related to alcohol. Factors such as peer pressure and social norms may contribute to mixed positive and negative expectancies. It is also plausible that while an adolescent may intellectually understand the negative consequences of alcohol, they may still be influenced by the immediate positive feelings related to alcohol use. As other studies have found higher health literacy to be associated with higher positive expectancies, higher parent education may be explained by similar mechanisms [3]. The present study found significant associations between higher household income and higher positive and negative alcohol expectancies, similar to previous literature outside the U.S. [17]. However, associations between higher income and higher positive expectancies were stronger and more consistent than associations between higher income and higher negative expectancies.

The current study has several limitations. The alcohol expectancy measures were self-reported, which increases the risk for social-desirability bias. Analyses are cross-sectional. Adolescents with more than one race/ethnicity were categorized as only one race/ethnic group in the analysis (e.g., Black non-Latino/Hispanic), which may not capture the nuances of their multiracial identities. However, the current study has several strengths including the large, diverse, nationwide sample that targeted early adolescents and examined a variety of relevant sociodemographic factors.

## Conclusions

The current study identified specific groups with demographic factors that may be protective against the development of risky alcohol attitudes. For example, Black non-Latino/Hispanic and Hispanic/Latino adolescents had less positive and negative alcohol expectancies. We identified specific groups that may be prone to developing risky alcohol attitudes. This includes older adolescents, sexual minority adolescents, or White non-Latino/Hispanic adolescents. The current study’s results indicate specific characteristics of early adolescents that imply susceptibility to future alcohol use based on their attitudes regarding alcohol. Our results offer useful implications within the realm of public health. Examples include school and community intervention programs that can change adolescent alcohol expectancies to reduce risk for and delay initiation of alcohol use [40, 41]. The current study also found novel associations between negative alcohol expectancies and several sociodemographic factors. As the interpretation of negative alcohol expectancies has differed between studies [42], future studies should further explore the role of negative alcohol expectancies in predicting future alcohol use. Future research should also examine the mechanisms between these sociodemographic factors and alcohol expectancy development.

### Electronic supplementary material

Below is the link to the electronic supplementary material.

Supplementary Material 1

Supplementary Material 2

## Data Availability

Data used in the preparation of this article were obtained from the ABCD Study (https://abcdstudy.org), held in the NIMH Data Archive (NDA).

## List of abbreviations

ABCD: Adolescent Brain Cognitive Development Study
AEQ-AB: Alcohol Expectancy Questionnaire- Adolescent, Brief
IRB: Institutional Review Board
UCSD: University of California, San Diego
