# Supplementary material for: Social epidemiology of early adolescent alcohol expectancies
Source: BMC Public Health. 2023 Dec 13;23:2502. doi: 10.1186/s12889-023-17434-5 (PMC10720177; doi:10.1186/s12889-023-17434-5)
Supplement: Supplementary file 1 — Supplementary Material 1 [file 12889_2023_17434_MOESM1_ESM.docx]

**Additional files:**

File name: Table S1

File format: .docx

Title of data: Comparison of participants non-missing vs missing

Description of data: Table

File name: Table S2

File format: .docx

Title of data: Alcohol Expectancy Questionnaire-Adolescent, Brief (AEQ-AB)

Description of data: Table

File name: Table S3

File format: .docx

Title of data: Detailed description of sociodemographic measures

Description of data: Table

File name: Table S4

File format: .docx

Title of data: Associations with alcohol sipping and positive and negative alcohol expectancies in the Adolescent Brain Cognitive Development (ABCD) Study (N=11,868)

Description of data: Table

File name: Table S5

File format: .docx

Title of data: Sociodemographic associations with positive alcohol expectancies in the Adolescent Brain Cognitive Development (ABCD) Study (N=11,868), stratified by sipping alcohol

Description of data: Table

File name: Table S6

File format: .docx

Title of data: Sociodemographic associations with negative alcohol expectancies in the Adolescent Brain Cognitive Development (ABCD) Study (N=11,868), stratified by sipping alcohol

Description of data: Table

File name: Table S7

File format: .docx

Title of data: Interaction tests with sociodemographic characteristics and sipping alcohol

Description of data: Table
