# Supplementary material for: Social epidemiology of early adolescent alcohol expectancies
Source: BMC Public Health. 2023 Dec 13;23:2502. doi: 10.1186/s12889-023-17434-5 (PMC10720177; doi:10.1186/s12889-023-17434-5)
Supplement: Supplementary file 2 — Supplementary Material 2 [file 12889_2023_17434_MOESM2_ESM.docx]

| Table S1. Comparison of participants with non-missing vs missing data | | | |
| --- | --- | --- | --- |
| Sociodemographic characteristics | Non-missing  (n= 9,017) | Missing  (n= 2,863) | p |
| Sex (%) |  |  | 0.706 |
| Female | 48.70% | 49.20% |  |
| Male | 51.30% | 50.80% |  |
| Race/ethnicity (%) |  |  | <0.001 |
| White non-Latino/Hispanic | 57.40% | 38.10% |  |
| Latino / Hispanic | 18.80% | 24.10% |  |
| Black non-Latino/Hispanic | 14.40% | 25.90% |  |
| Asian non-Latino/Hispanic | 5.20% | 6.40% |  |
| Native American non-Latino/Hispanic | 3.00% | 3.60% |  |
| Other non-Latino/Hispanic | 1.30% | 2.00% |  |
| Sexual minority (%) |  |  | 0.074 |
| No | 73.50% | 75.80% |  |
| Yes or questioning | 1.10% | 1.40% |  |
| Don't understand the question | 23.10% | 25.10% |  |
| Household income (%) |  |  | <0.001 |
| $75,000 and greater | 54.00% | 26.20% |  |
| Less than $75,000 | 46.00% | 73.80% |  |
| Parents' highest education (%) |  |  | <0.001 |
| College education or more | 86.60% | 67.40% |  |
| High school education or less | 13.40% | 32.60% |  |
| Parent marital status (%) |  |  | <0.001 |
| Married/partnered | 71.80% | 57.40% |  |
| Not married/unpartnered/single | 28.20% | 42.60% |  |
| Religiosity (%) |  |  | 0.942 |
| Religious | 74.60% | 74.50% |  |
| Not religious | 25.40% | 25.50% |  |
| ABCD propensity weights were applied based on the American Community Survey from the US Census. | | | |

Table S2. Alcohol Expectancy Questionnaire-Adolescent, Brief (AEQ-AB)

| Variable name | Items | Response Options | Expectancy |
| --- | --- | --- | --- |
| aeq_section_q01 | Alcohol helps a person relax, feel happy, feel less tense, and can keep a person's mind off of mistakes at school or work. | 1=Disagree Strongly; 2=Disagree Somewhat; 3=Uncertain; 4=Agree Somewhat; 5=Agree Strongly | Positive |
| aeq_section_q02 | Alcohol can help how well a person gets along with others (makes people want to have fun together). | 1=Disagree Strongly; 2=Disagree Somewhat; 3=Uncertain; 4=Agree Somewhat; 5=Agree Strongly | Positive |
| aeq_section_q03 | Alcohol can hurt how well a person gets along with others (makes people mean to others). | 1=Disagree Strongly; 2=Disagree Somewhat; 3=Uncertain; 4=Agree Somewhat; 5=Agree Strongly | Negative |
| aeq_section_q04 | Alcohol helps people think better and helps coordination (people understand things better; can do things better). | 1=Disagree Strongly; 2=Disagree Somewhat; 3=Uncertain; 4=Agree Somewhat; 5=Agree Strongly | Positive |
| aeq_section_q05 | Alcohol hurts how people think and it hurts their coordination (run into things, act silly, have a hangover). | 1=Disagree Strongly; 2=Disagree Somewhat; 3=Uncertain; 4=Agree Somewhat; 5=Agree Strongly | Negative |
| aeq_section_q06 | Alcohol makes a person feel stronger and more powerful (easier to fight, speak in front of others, stand up to others). | 1=Disagree Strongly; 2=Disagree Somewhat; 3=Uncertain; 4=Agree Somewhat; 5=Agree Strongly | Positive |
| aeq_section_q07 | Alcohol can make people more careless or do things that could get them into trouble (do things they feel bad about; do things they regret). | 1=Disagree Strongly; 2=Disagree Somewhat; 3=Uncertain; 4=Agree Somewhat; 5=Agree Strongly | Negative |

Table S3. Detailed description of sociodemographic measures

| **Sex.** Parents reported the sex assigned at birth of the child (female or male). |
| --- |
| **Race/ethnicity.** Parents were asked at baseline, “What race do you consider the child to be? Please check all that apply.” Response options included: White, Black/African American, American Indian/Native American, Alaska Native, Asian Indian, Chinese, Filipino, Japanese, Korean, Vietnamese, Other Asian, Native Hawaiian, Guamanian, Samoan, Other Pacific Islander, and Other race. American Indian/Native American and Alaska Native were combined into a single category. Asian Indian, Chinese, Filipino, Japanese, Korean, Vietnamese, Other Asian, Native Hawaiian, Guamanian, Samoan, and Other Pacific Islander, were combined into an Asian/Pacific Islander category. To assess ethnicity, parents were additionally asked, “Do you consider the child Hispanic/Latino/Latina?” Based on these questions, a single race/ethnicity variable was constructed following guidance from the National Longitudinal Study of Adolescent to Adult Health: White non-Latino/Hispanic, Latino/Hispanic, Black non-Latino/Hispanic, Asian non-Latino/Hispanic, Native American non-Latino/Hispanic, and other non-Latino/Hispanic [26]. |
| **Household income.** Parents were asked in Year 2, “What is your TOTAL COMBINED FAMILY INCOME for the past 12 months? This should include income (before taxes and deductions) from all sources, wages, rent from properties, social security, disability and/or veteran's benefits, unemployment benefits, workman's compensation, help from relative (include child payments and alimony), and so on.” Response options included: Less than $5,000; $5,000 through $11,999; $12,000 through $15,999; $16,000 through $24,999; $25,000 through $34,999; $35,000 through $49,999; $50,000 through $74,999; $75,000 through $99,999; $100,000 through $199,999; $200,000 and greater. Given small sample sizes for some of the options, income was categorized into six categories: less than $25,000, $25,000 through $49,999, $50,000 through $74,999, $75,000 through $99,999, $100,000 through $199,999, $200,000 and greater. |
| **Highest parent education.** Parents were asked in Year 2, “What is the highest grade or level of school you have completed or the highest degree you have received?” and “What is the highest grade or level of school your partner completed or highest degree they received?” The survey clarifies the definition of a partner, “Your "partner" refers to any significant figure in your life that helps you in raising your child or has helped you for more than 2 years. This person should be involved 40% or more of the daily activities your child does. For example, your partner could be your spouse. However, your partner could also be your boyfriend/girlfriend or relative.” Response options included: Never attended/Kindergarten only, 1st grade, 2nd grade, 3rd grade, 4th grade, 5th grade, 6th grade, 7th grade, 8th grade, 9th grade, 10th grade, 11th grade, 12th grade, High school graduate, GED or equivalent, Some college, Associate degree: Occupational, Associate degree: Academic Program, Bachelor's degree (ex. BA), Master's degree (ex. MA), Professional School degree (ex. MD), Doctoral degree (ex. PhD). The highest level of education of the parent or partner was used for this analysis. Response categories were combined into high school or less (Never attended/Kindergarten only, 1st grade, 2nd grade, 3rd grade, 4th grade, 5th grade, 6th grade, 7th grade, 8th grade, 9th grade, 10th grade, 11th grade, 12th grade, High school graduate, GED or equivalent) and college education or more (Some college, Associate degree: Occupational, Associate degree: Academic Program, Bachelor's degree, Master's degree, Professional School degree, Doctoral degree). |
| **Parent marital status.** Parents were asked in Year 2, “Are you now married, widowed, divorced, separated, never married or living with a partner?” Response options included: married, widowed, divorced, separated, never married, or living with partner. Married and living with a partner were combined. Widowed, divorced, separated, and never married were categorized as not married/not living with a partner. |
| **Sexual orientation.** Participants were asked in Year 2, “Are you gay or bisexual?” Response options included: yes, maybe, no, and don't understand the question. |
| **Child religiosity.** Parents were asked at baseline, “What is the child's religious preference?” Response options included: Mainline Protestant, Evangelical Protestant, Historically Black Church, Roman Catholic (Catholic), Jewish (Judaism), Mormon (Church of Jesus Christ of Latter Day Saints/LDS), Jehovah's Witness, Muslim (Islam), Buddhist, Hindu, Orthodox Christian, Unitarian (Universalist), Other Christian, Atheist (do not believe in God), Agnostic (not sure if there is a God), Something else, Nothing in Particular. Responses were combined into religious (Mainline Protestant, Evangelical Protestant, Historically Black Church, Roman Catholic, Jewish, Mormon, Jehovah's Witness, Muslim, Buddhist, Hindu, Orthodox Christian, Unitarian, Other Christian) and not religious (Atheist, Agnostic, Something else, Nothing in Particular). |

| Table S4. Associations with alcohol sipping and positive and negative alcohol expectancies in the Adolescent Brain Cognitive Development (ABCD) Study (N=11,868) | | | | |
| --- | --- | --- | --- | --- |
|  | Positive Alcohol Expectancies, Adjusted | | Negative Alcohol Expectancies, Adjusted | |
|  | β (95% CI) | p | β (95% CI) | p |
| Prior alcohol sipping |  |  |  |  |
| No | reference |  | reference |  |
| Yes | **0.35 (0.28, 0.42)** | **<0.001** | 0.05 (-0.01, 0.12) | 0.110 |
| Age (years) | **0.11 (0.09, 0.13)** | **<0.001** | **0.03 (0.01, 0.05)** | **0.004** |
| Sex |  |  |  |  |
| Female | reference |  | reference |  |
| Male | **0.03 (-0.01, 0.08)** | **0.106** | **0.07 (0.03, 0.11)** | **0.002** |
| Race/ethnicity |  |  |  |  |
| White non-Latino/Hispanic | reference |  | reference |  |
| Latino / Hispanic | **-0.08 (-0.14, -0.02)** | **0.008** | **-0.09 (-0.16, -0.02)** | **0.015** |
| Black non-Latino/Hispanic | **-0.10 (-0.17, -0.03)** | **0.005** | **-0.09 (-0.16, -0.03)** | **0.003** |
| Asian non-Latino/Hispanic | -0.06 (-0.16, 0.04) | 0.237 | -0.01 (-0.12, 0.10) | 0.837 |
| Native American non-Latino/Hispanic | -0.08 (-0.20, 0.04) | 0.174 | -0.10 (-0.21, 0.02) | 0.097 |
| Other non-Latino/Hispanic | -0.10 (-0.26, 0.07) | 0.244 | -0.10 (-0.27, 0.07) | 0.255 |
| Sexual minority status |  |  |  |  |
| No | reference |  | reference |  |
| Yes | **0.28 (0.18, 0.37)** | **<0.001** | **0.13 (0.04, 0.23)** | **0.008** |
| Maybe | **0.24 (0.14, 0.34)** | **<0.001** | 0.08 (-0.03, 0.19) | 0.134 |
| Don't understand the question | -0.07 (-0.18, 0.05) | 0.251 | -0.06 (-0.18, 0.06) | 0.319 |
| Decline to answer | 0.10 (-0.08, 0.27) | 0.272 | 0.10 (-0.07, 0.27) | 0.260 |
| Household income |  |  |  |  |
| $200,000 and greater | reference |  | reference |  |
| $100,000 to $199,999 | **-0.10 (-0.19, -0.02)** | **0.012** | 0.00 (-0.08, 0.08) | 0.982 |
| $75,000 to $99,999 | -0.09 (-0.18, 0.00) | 0.053 | -0.04 (-0.13, 0.05) | 0.361 |
| $50,000 to $74,999 | **-0.12 (-0.21, -0.03)** | **0.008** | -0.09 (-0.18, 0.00) | 0.056 |
| $25,000 to $49,999 | **-0.11 (-0.21, -0.02)** | **0.018** | -0.05 (-0.14, 0.04) | 0.294 |
| $24,999 or less | **-0.15 (-0.25, -0.05)** | **0.005** | **-0.18 (-0.28, -0.09)** | **<0.001** |
| Parent's highest education |  |  |  |  |
| College education or more | reference |  | reference |  |
| High school education or less | **-0.11 (-0.18, -0.05)** | **<0.001** | **-0.21 (-0.28, -0.15)** | **<0.001** |
| Parents’ marital status |  |  |  |  |
| Married/partnered | reference |  | reference |  |
| Not married/unpartnered/single | **0.05 (0.00, 0.10)** | **0.040** | 0.02 (-0.03, 0.07) | 0.494 |
| Child religiosity |  |  |  |  |
| Religious | reference |  | reference |  |
| Not religious | **0.13 (0.09, 0.18)** | **<0.001** | 0.01 (-0.04, 0.05) | 0.785 |
| Bold indicates p<0.05. ABCD propensity weights were applied based on the American Community Survey from the US Census. All models include age, sex, race/ethnicity, sexual orientation, household income, parent education, parent marital status, child religiosity, and study site. | | | | |

| Table S5. Sociodemographic associations with positive alcohol expectancies in the Adolescent Brain Cognitive Development (ABCD) Study (N=11,868), stratified by sipping alcohol | | | | |
| --- | --- | --- | --- | --- |
|  | Positive Alcohol Expectancies | | | |
|  | Never sipped alcohol | | Sipped alcohol | |
| Sociodemographic characteristics | β (95% CI) | p | β (95% CI) | p |
| Age (years) | **0.11 (0.09, 0.13)** | **<0.001** | **0.16 (0.10, 0.23)** | **<0.001** |
| Sex |  |  |  |  |
| Female | reference |  | reference |  |
| Male | **0.05 (0.01, 0.09)** | **0.025** | 0.00 (-0.13, 0.13) | 0.972 |
| Race/ethnicity |  |  |  |  |
| White non-Latino/Hispanic | reference |  | reference |  |
| Latino / Hispanic | -0.07 (-0.13, 0.00) | 0.061 | **-0.33 (-0.55, -0.11)** | **0.003** |
| Black non-Latino/Hispanic | **-0.11 (-0.18, -0.05)** | **<0.001** | -0.05 (-0.30, 0.21) | 0.716 |
| Asian non-Latino/Hispanic | -0.05 (-0.15, 0.04) | 0.269 | -0.17 (-0.46, 0.11) | 0.236 |
| Native American non-Latino/Hispanic | -0.10 (-0.22, 0.02) | 0.092 | 0.10 (-0.30, 0.51) | 0.614 |
| Other non-Latino/Hispanic | -0.02 (-0.20, 0.15) | 0.787 | **-0.98 (-1.5, -0.49)** | **<0.001** |
| Sexual minority status |  |  |  |  |
| No | reference |  | reference |  |
| Yes | **0.34 (0.24, 0.45)** | **<0.001** | **0.26 (0.03, 0.49)** | **0.026** |
| Maybe | **0.30 (0.19, 0.41)** | **<0.001** | 0.22 (-0.02, 0.46) | 0.073 |
| Don't understand the question | -0.09 (-0.20, 0.03) | 0.133 | -0.06 (-0.53, 0.40) | 0.789 |
| Decline to answer | **0.21 (0.02, 0.39)** | **0.028** | **-0.53 (-1.0, -0.07)** | **0.025** |
| Household income |  |  |  |  |
| $200,000 and greater | reference |  | reference |  |
| $100,000 to $199,999 | **-0.13 (-0.22, -0.04)** | **0.006** | **-0.06 (-0.27, 0.16)** | **0.591** |
| $75,000 to $99,999 | -0.10 (-0.19, 0.00) | 0.053 | -0.03 (-0.27, 0.21) | 0.816 |
| $50,000 to $74,999 | **-0.15 (-0.24, -0.05)** | **0.003** | -0.01 (-0.25, 0.23) | 0.959 |
| $25,000 to $49,999 | **-0.14 (-0.24, -0.04)** | **0.007** | 0.09 (-0.17, 0.35) | 0.507 |
| $24,999 or less | **-0.16 (-0.27, -0.06)** | **0.003** | -0.04 (-0.40, 0.31) | 0.818 |
| Parent's highest education |  |  |  |  |
| College education or more | reference |  | reference |  |
| High school education or less | **-0.12 (-0.18, -0.06)** | **<0.001** | -0.15 (-0.43, 0.13) | 0.287 |
| Parents’ marital status |  |  |  |  |
| Married/partnered | reference |  | reference |  |
| Not married/unpartnered/single | 0.05 (0.00, 0.10) | 0.056 | 0.01 (-0.15, 0.17) | 0.907 |
| Child religiosity |  |  |  |  |
| Religious | reference |  | reference |  |
| Not religious | **0.12 (0.07, 0.17)** | **<0.001** | 0.12 (-0.02, 0.26) | 0.094 |
| Bold indicates p<0.05. ABCD propensity weights were applied based on the American Community Survey from the US Census. All models include age, sex, race/ethnicity, sexual orientation, household income, parent education, parent marital status, child religiosity, and study site. | | | | |

| Table S6. Sociodemographic associations with negative alcohol expectancies in the Adolescent Brain Cognitive Development (ABCD) Study (N=11,868), stratified by sipping alcohol | | | | |
| --- | --- | --- | --- | --- |
|  | Negative Alcohol Expectancies | | | |
|  | Never sipped alcohol | | Sipped alcohol | |
| Sociodemographic characteristics | β (95% CI) | p | β (95% CI) | p |
| Age (years) | **0.03 (0.01, 0.05)** | **0.008** | 0.02 (-0.05, 0.08) | 0.644 |
| Sex |  |  |  |  |
| Female | reference |  | reference |  |
| Male | **0.07 (0.03, 0.11)** | **<0.001** | 0.08 (-0.05, 0.22) | 0.220 |
| Race/ethnicity |  |  |  |  |
| White non-Latino/Hispanic | reference |  | reference |  |
| Latino / Hispanic | **-0.11 (-0.18, -0.04)** | **0.002** | -0.07 (-0.29, 0.15) | 0.538 |
| Black non-Latino/Hispanic | **-0.12 (-0.18, -0.05)** | **<0.001** | -0.03 (-0.30, 0.23) | 0.807 |
| Asian non-Latino/Hispanic | 0.00 (-0.10, 0.10) | 0.971 | 0.02 (-0.28, 0.32) | 0.893 |
| Native American non-Latino/Hispanic | -0.12 (-0.24, 0.01) | 0.068 | 0.01 (-0.41, 0.42) | 0.977 |
| Other non-Latino/Hispanic | -0.16 (-0.34, 0.02) | 0.076 | 0.35 (-0.15, 0.86) | 0.172 |
| Sexual minority status |  |  |  |  |
| No | reference |  | reference |  |
| Yes | **0.16 (0.05, 0.27)** | **0.003** | 0.17 (-0.07, 0.40) | 0.163 |
| Maybe | 0.10 (-0.02, 0.21) | 0.095 | 0.17 (-0.08, 0.42) | 0.173 |
| Don't understand the question | -0.03 (-0.15, 0.09) | 0.631 | **-0.73 (-1.2, -0.25)** | **0.003** |
| Decline to answer | 0.14 (-0.05, 0.33) | 0.152 | -0.16 (-0.64, 0.31) | 0.499 |
| Household income |  |  |  |  |
| $200,000 and greater | reference |  | reference |  |
| $100,000 to $199,999 | 0.00 (-0.10, 0.09) | 0.932 | 0.03 (-0.19, 0.24) | 0.803 |
| $75,000 to $99,999 | -0.03 (-0.14, 0.07) | 0.522 | 0.00 (-0.25, 0.25) | 0.977 |
| $50,000 to $74,999 | -0.08 (-0.18, 0.02) | 0.119 | -0.09 (-0.34, 0.16) | 0.493 |
| $25,000 to $49,999 | -0.04 (-0.15, 0.07) | 0.459 | 0.07 (-0.21, 0.35) | 0.627 |
| $24,999 or less | **-0.18 (-0.29, -0.06)** | **0.002** | **-0.41 (-0.79, -0.02)** | **0.039** |
| Parent's highest education |  |  |  |  |
| College education or more | reference |  | reference |  |
| High school education or less | **-0.20 (-0.27, -0.14)** | **<0.001** | -0.28 (-0.57, 0.01) | 0.058 |
| Parents’ marital status |  |  |  |  |
| Married/partnered | reference |  | reference |  |
| Not married/unpartnered/single | 0.04 (-0.02, 0.09) | 0.177 | -0.06 (-0.23, 0.11) | 0.481 |
| Child religiosity |  |  |  |  |
| Religious | reference |  | reference |  |
| Not religious | 0.00 (-0.05, 0.05) | 0.907 | 0.07 (-0.08, 0.21) | 0.353 |
| Bold indicates p<0.05. ABCD propensity weights were applied based on the American Community Survey from the US Census. All models include age, sex, race/ethnicity, sexual orientation, household income, parent education, parent marital status, child religiosity, and study site. | | | | |

| Table S7. Interaction tests with sociodemographic characteristics and sipping alcohol | | | | |
| --- | --- | --- | --- | --- |
|  | Positive Expectancies | | Negative Expectancies | |
| Sociodemographic characteristics | β (95% CI) | p | β (95% CI) | p |
| Age (years) | 0.03 (-0.04, 0.10) | 0.361 | -0.01 (-0.07, 0.06) | 0.877 |
| Sex |  |  |  |  |
| Female | reference | reference | reference | reference |
| Male | -0.02 (-0.14, 0.11) | 0.789 | -0.02 (-0.16, 0.11) | 0.748 |
| Race/ethnicity |  |  |  |  |
| White non-Latino/Hispanic | reference | reference | reference | reference |
| Latino / Hispanic | -0.13 (-0.33, 0.07) | 0.195 | 0.00 (-0.19, 0.19) | 0.995 |
| Black non-Latino/Hispanic | 0.09 (-0.14, 0.31) | 0.447 | 0.06 (-0.22, 0.33) | 0.674 |
| Asian non-Latino/Hispanic | -0.13 (-0.43, 0.17) | 0.391 | -0.02 (-0.32, 0.28) | 0.886 |
| Native American non-Latino/Hispanic | 0.16 (-0.25, 0.58) | 0.435 | 0.11 (-0.32, 0.53) | 0.620 |
| Other non-Latino/Hispanic | **-0.83 (-1.40, -0.29)** | **0.003** | 0.52 (0.00, 1.00) | 0.051 |
| Sexual minority status |  |  |  |  |
| No | reference | reference | reference | reference |
| Yes | 0.02 (-0.22, 0.26) | 0.865 | -0.01 (-0.26, 0.24) | 0.941 |
| Maybe | 0.04 (-0.22, 0.29) | 0.785 | 0.05 (-0.25, 0.35) | 0.755 |
| Don't understand the question | 0.04 (-0.41, 0.50) | 0.854 | **-0.49 (-0.96, -0.01)** | **0.044** |
| Decline to answer | **-0.56 (-1.10, -0.01)** | **0.046** | -0.24 (-0.75, 0.26) | 0.342 |
| Household income |  |  |  |  |
| $200,000 and greater | reference | reference | reference | reference |
| $100,000 to $199,999 | 0.05 (-0.18, 0.28) | 0.651 | 0.03 (-0.19, 0.25) | 0.779 |
| $75,000 to $99,999 | 0.04 (-0.21, 0.29) | 0.764 | 0.04 (-0.22, 0.29) | 0.773 |
| $50,000 to $74,999 | 0.09 (-0.15, 0.34) | 0.446 | 0.02 (-0.23, 0.28) | 0.857 |
| $25,000 to $49,999 | 0.13 (-0.13, 0.39) | 0.317 | 0.10 (-0.15, 0.34) | 0.441 |
| $24,999 or less | 0.04 (-0.27, 0.34) | 0.812 | -0.14 (-0.47, 0.20) | 0.415 |
| Parent's highest education |  |  |  |  |
| College education or more | reference | reference | reference | reference |
| High school education or less | -0.01 (-0.26, 0.24) | 0.929 | -0.02 (-0.29, 0.24) | 0.865 |
| Parents’ marital status |  |  |  |  |
| Married/partnered | reference | reference | reference | reference |
| Not married/unpartnered | -0.02 (-0.16, 0.13) | 0.812 | -0.08 (-0.26, 0.10) | 0.362 |
| Child religiosity |  |  |  |  |
| Religious | reference | reference | reference | reference |
| Not religious | -0.01 (-0.16, 0.14) | 0.891 | 0.06 (-0.08, 0.20) | 0.414 |
| Bold indicates p<0.05. ABCD propensity weights were applied based on the American Community Survey from the US Census. All models include age, sex, race/ethnicity, sexual orientation, household income, parent education, parent marital status, child religiosity, and study site. | | | | |
